# Inferring the Transcriptional Landscape of Bovine Skeletal Muscle by Integrating Co-Expression Networks

**DOI:** 10.1371/journal.pone.0007249

**Published:** 2009-10-01

**Authors:** Nicholas J. Hudson, Antonio Reverter, YongHong Wang, Paul L. Greenwood, Brian P. Dalrymple

**Affiliations:** 1 Food Futures Flagship and Livestock Industries, Commonwealth Scientific and Industrial Research Organisation, Queensland Bioscience Precinct, St. Lucia, Brisbane, Queensland, Australia; 2 NSW (New South Wales) Department of Primary Industries Beef Industry Centre of Excellence, University of New England, Armidale, New South Wales, Australia; Fondazione Telethon, Italy

## Abstract

**Background:**

Despite modern technologies and novel computational approaches, decoding causal transcriptional regulation remains challenging. This is particularly true for less well studied organisms and when only gene expression data is available. In muscle a small number of well characterised transcription factors are proposed to regulate development. Therefore, muscle appears to be a tractable system for proposing new computational approaches.

**Methodology/Principal Findings:**

Here we report a simple algorithm that asks “which transcriptional regulator has the highest average absolute co-expression correlation to the genes in a co-expression module?” It correctly infers a number of known causal regulators of fundamental biological processes, including cell cycle activity (E2F1), glycolysis (HLF), mitochondrial transcription (TFB2M), adipogenesis (PIAS1), neuronal development (TLX3), immune function (IRF1) and vasculogenesis (SOX17), within a skeletal muscle context. However, none of the canonical pro-myogenic transcription factors (MYOD1, MYOG, MYF5, MYF6 and MEF2C) were linked to muscle structural gene expression modules. Co-expression values were computed using developing bovine muscle from 60 days post conception (early foetal) to 30 months post natal (adulthood) for two breeds of cattle, in addition to a nutritional comparison with a third breed. A number of transcriptional landscapes were constructed and integrated into an always correlated landscape. One notable feature was a ‘metabolic axis’ formed from glycolysis genes at one end, nuclear-encoded mitochondrial protein genes at the other, and centrally tethered by mitochondrially-encoded mitochondrial protein genes.

**Conclusions/Significance:**

The new module-to-regulator algorithm complements our recently described Regulatory Impact Factor analysis. Together with a simple examination of a co-expression module's contents, these three gene expression approaches are starting to illuminate the *in vivo* transcriptional regulation of skeletal muscle development.

## Introduction

We are interested in addressing the transcriptional regulatory rewiring that underpins muscle development and evolution. However, such an approach is predicated on first having a basic understanding of the core, conserved relationships that exist between genes within a single muscle and species. In order to achieve these aims, we have chosen the bovine *longissimus dorsi* muscle as our model system. Unlike rodent laboratory models [1], a large animal system such as the bovine allows reliable identification of skeletal muscle even in the very early pre-natal stages (primary, secondary and tertiary myogenesis). In turn, this permits a developmental sequence not experimentally feasible in other mammals. Moreover, the bovine is arguably a superior biomedical model than the rodent because 1) its protein sequences are more similar; 2) some genetic disorders of relevance to humans are heritable in bovine and not in rodents; and 3) their larger size makes bovines closer to humans from a biomechanical perspective.

Differences in transcriptional regulation underpin much biological variation, from cellular responses within a few minutes to evolutionary change over eons [2]–[4]. Under the correct cellular circumstances, Transcription Factors (TF's), in coordination with transcriptional co-factors, ligands, the appropriate signalling cascade and a receptive chromatin structure, will bind to a target gene's promoter region culminating in a targeted gene expression response. Despite a combination of modern technologies such as high density single nucleotide polymorphism (SNP) panels, transcriptional profiling, ChIP-on-chip data [5], [6], together with computational approaches including eQTL [7], eQED [8], Regulatory Potential [9] and Regulatory Impact Factors [10], decoding causal transcriptional regulation remains a challenge. For example, the application of ChIP-on-Chip across a wide diversity of TF's and species is lagging well behind the generation of gene expression data.

Networks are a promising tool for modelling, analysis and visualisation, and are considered semi-quantitative graphical representations of transcriptional regulation. Their topology reveals modules (clusters of functionally related genes and their regulators) and hubs (genes with high transcriptional connectivity) in a non-random fashion often characterized by a connectivity structure that follows a scale-free power-law distribution [11]. One method for building biological networks is to establish connections (edges) between genes (nodes) whose expression profiles are significantly correlated. While there are numerous such co-expression networks reported in the literature [12]–[16], the only other muscle-specific network is much sparser (comprising 822 genes and 26 TF) [17]. To maximise the robustness of this muscle network, we took advantage of two unique experimental resources for *in vivo* mammalian skeletal muscle biology [18]–[20] which together comprise 26 experimental treatments and 3 major perturbations (genetic, ontogenetic and nutrigenomic) within a single tissue and species. To the best of our knowledge, there is no tissue and species-specific developmental data set in the public domain that matches it for biological comprehensiveness.

A number of refinements to the analysis of gene expression correlation networks have been proposed for the identification of TF's controlling gene expression, including the incorporation of TF binding sites. However, many transcriptional regulators do not bind directly to DNA and, for many that do bind, the binding site is unknown [21], [22]. For example, the current release of MatBase (version 8.0) contains 1,751 human TF for which there is a position weight matrix description of the binding site for only 728. In addition, the binding sites of many TFs are so similar that they do not allow a reliable prediction of function; clearly, sequence preferences can be altered by the binding context [23]. Fundamentally, the biological processes mediated by many TF are unknown [21]. This raises an important question; can we identify complementary genomic approaches that help infer TF regulation but do not require binding site data?

Here we describe the application of PCIT [24] to construct a mammalian muscle gene expression correlation network. Additionally, we describe a new method that helps infer the transcriptional regulators involved in the regulation of the various network modules. Because we exclusively focussed on one tissue type, the inviolable modules of mammalian life and their transcriptional regulation are captured within a muscle-specific context. The output thus represents a powerful functional genomic information resource for mammalian myobiology, and should generate robust hypotheses for a host of downstream *in vitro* and *in vivo* validations. In light of our regulatory findings, we briefly discuss the limitations as well as the promise of co-expression approaches.

## Materials and Methods

### Ethics statement

Use of animals and the procedures performed in this study was approved by the New South Wales North Coast and Animal Care and Ethics Committee (Approval no. G2000/05).

### Tissues sampled

Details regarding animal resource and experimental designs can be found in the recent literature [18]–[20]. In brief, *longissimus dorsi* skeletal muscle biopsies of Piedmontese cross Hereford and Wagyu cross Hereford *Bos taurus* cattle were acquired at 10 developmental time points (3 pre-natal, birth and 6 post-natal) and from tropically-adapted Belmont Red cattle (*Bos taurus*: 50% Afrikander, 25% Hereford and 25% shorthorn) throughout a nutritional deprivation and re-alimentation experiment comprising 3 adult time points for each of the two treatments. RNA was extracted as previously described [18]–[20].

### Computing the Always Correlated Network

Unlike these previously described studies which used a much sparser cDNA platform, these transcriptomes were assayed using a bovine oligonucleotide microarray, developed in 2006 by ViaLactia Bioscience in collaboration with Agilent, containing 21,475 unique 60-mer probes, representing approximately 19,500 distinct bovine genes. Preliminary edits resulted in 6,077 probes being discarded because they were expressed at levels below the sensitivity of the platform (i.e., negative signal to noise ratio) in all hybridizations. Of the remaining 15,398 probes, 11,421 different genes were represented capturing ∼52% of the 22,000 total genes estimated by the recent bovine sequencing effort [25]. An additional 1,673 probes were found to have dubious gene assignments (due, for example, to identical matches to multiple genes). The 13,094 potential ‘nodes’ were then pre-filtered, with all genes whose expression showed no significant deviation from the mean, defined by a one standard deviation interval, across any of the 26 treatments removed in an effort to minimise spurious correlations. These editing criteria resulted in a total of 6,603 genes for which detectably strong and variable expression across treatments was available.

**Figure 1:**
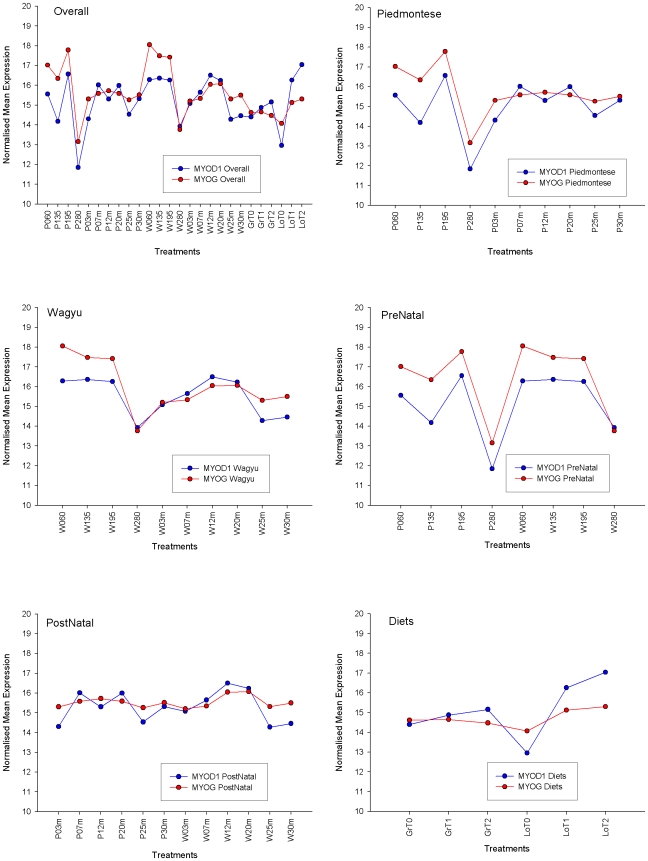
The profiles of MYOD1 and MYOG across the 6 transcriptional landscapes. Their significant correlation in each of the 6 instances explains their inclusion in the Always Correlated landscape.

We computed correlation co-efficients among each of 6,603 genes and reverse-engineered transcriptional networks using PCIT [24]: In brief, PCIT belongs to the family of weighted network algorithms and works by comparing the co-expression arrangements for triplets of genes, with all triplets being exhaustively explored. To ascertain the strength of mutual independence, for each triplet the co-expression between two members that can be attributed to the correlation to the 3^rd^ member is determined. This approach helps discard spurious correlation co-efficients i.e. arrangements where a large proportion of the correlation between two genes is actually attributable to the presence of a third gene. Given that the relationship between each triplet is different, significance can occur at a range of correlation coefficients. An edge was only formed in the ‘Always Correlated’ transcriptional landscape if a significant correlation persisted between the expression of that particular pair of genes across all six landscapes i.e. irrespective of the animal's genetic background, developmental stage and nutritional status. The sign on the edge in the Always Correlated landscape was taken from the Overall landscape. The Overall landscape is based on the significant correlations computed across all 26 treatments (as denoted on the x-axis of Figure 1, top left panel). The correlations for the Overall landscape were built from all the experiments laid end-to-end i.e. the full Piedmontese development time course (10 time points), the full Wagyu development time course (10 time points) and the nutritional restriction experiment (6 time points).

### The Transcriptional Regulators

For the purposes of mapping the Always Correlated transcriptional landscape we identified a broad range of transcriptional regulators, including not only TFs but also signalling molecules and chromatin remodellers, as has previously been published in a related context [26]. The *bona fide* TFs (sequence specific DNA binding factors) on the array platform were identified in two steps using Genomatix software (http://www.genomatix.de/). In the first step, the list of all the known gene names (HUGO) for the human was used as an input for Bibliosphere [27] and a list of TFs retrieved based on literature, gene ontology (GO) and manual annotation. In the second step, genome-wide searches for TFs were identified in MatBase [28] (based on TF matrices) for human. Subsequently, these two lists were collated and duplicate TF entries were removed to generate a final non-redundant list of 1,017. This list of TF is conservative as the full repertoire of putative TF in mammalian genomes is larger than documented here. For example, the Zinc Finger motifs number into the hundreds on their own.

The signalling molecules and chromatin remodelers groups were established based on GO terms following [26]. In brief, we examined files available at ftp://ftp.ncbi.nih.gov/gene/DATA/ which were obtained and searched by accession number to identify gene ontology information for each sequence. We then text searched for the following strings: “chromatin” and “signal.” (see Table S1 for identity of genes in these groups). It is possible that the ‘signal’ text search is not discriminatory enough to exclusively identify transcriptional regulators and this should be borne in mind when viewing the network.

### Module-to-Regulator Analysis

We identified modules of co-expressed genes in *Cytoscape 2.5.1* [29] using the organic clustering algorithm. The organic clustering option groups together genes with common neighbours. We then computed a downstream analysis which asks the question “which TF had the highest average absolute correlation to all the ‘target’ genes present in a given module?” In the event of a TF being a member of a module, it would be deemed a ‘target’ in this context. For the purposes of computing this correlation we used the ‘Overall’ contrast. The absolute correlation coefficients (i.e. unsigned) were used to avoid the problem of modules which contained a mix of positive and negative correlations.

In the instances where modules are continuous with another part of a network, an objective delineation of their component genes is not immediately apparent. Our resolution was to compute a more stringent (less connected and hence less cohesive) version of the landscape by only considering edges derived from significant PCIT connections and with correlations >0.85 in absolute value (data not shown). We then expanded the module of interest by adding on to its members only direct neighbours of the full PCIT set (see Table S2 for module genes).

This filtering procedure gave rise to separate networks for the main modules in the landscape which could then be used to more objectively identify the ‘target’ genes for the downstream module-to-regulator analysis. Some of the less cohesive modules (including the slow twitch module) were not maintained by this analysis and so do not feature in the output. Clearly, no filtering is necessary for those genes that are in discrete networks within the Always Correlated landscape, such as the vasculature, ribosome and fat modules. Of these, only modules containing at least 4 nodes were included in the analysis (with the sole exception of a fat module containing 3 nodes).

## Results

### The Always Correlated transcriptional landscape

**Figure 2:**
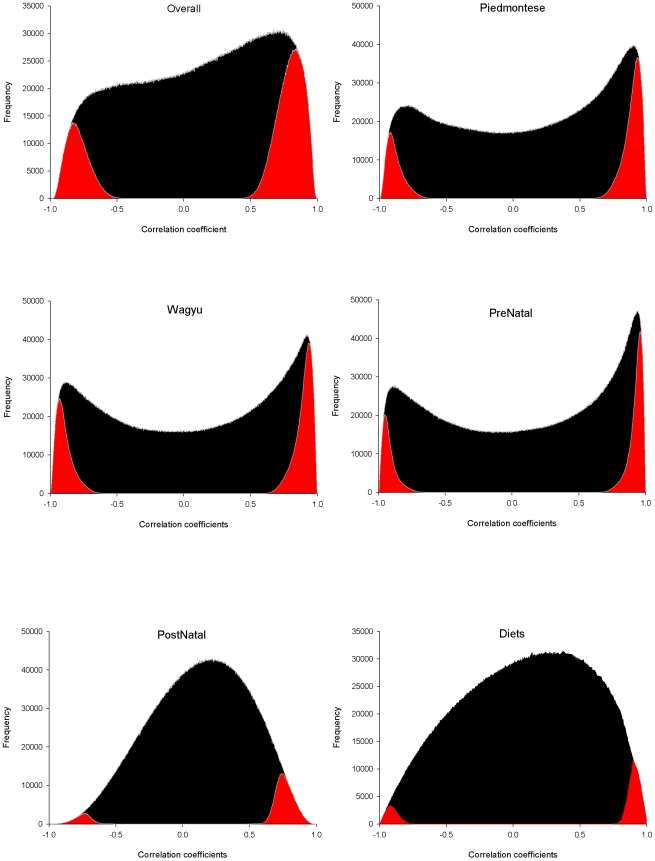
The frequency distributions of all correlation coefficients in each of the six transcriptional landscapes (black) plus those deemed significant by PCIT (red).

**Figure 3:**
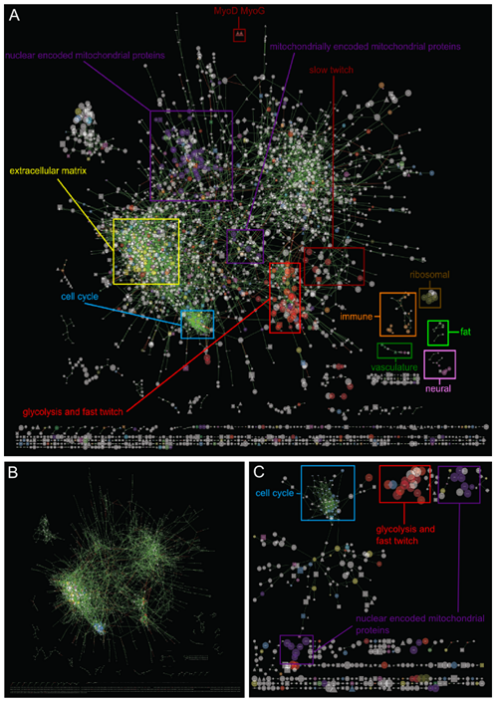
The Always Correlated transcriptional landscape. Networks were visualised using the organic algorithm of *Cytoscape* [29]. A) Node size was mapped to average transcript abundance, edge colour was mapped to the sign of the correlation in the “Overall” landscape and node colour was mapped to Gene Ontology process. Node shape was mapped as follows: TFs (triangles), signalling molecules (squares) and chromatin remodelers (diamonds). All other genes (i.e. non-regulators) were mapped as ovals. B) Node size was mapped to number of connections. C) The transcription landscape built from connections with correlation coefficients >0.99.

With the available 6,603 genes, we reverse-engineered the following six interlaced transcriptional landscapes using PCIT [24]: Overall (using all 26 experimental conditions), Piedmontese (10), Wagyu (10), Prenatal (8), Postnatal (12) and Nutrition (6). An appealing numerical feature of PCIT is that, irrespective of the overall distribution of correlation coefficients (which may or may not be normally distributed) the significant ones always follow a bell-shaped normal-like distribution (Figure 2). An edge in the Always Correlated transcription landscape was assigned when a significant PCIT correlation was observed for a pair of genes in all 6 parent transcriptional landscapes described above (Figure 1). Significant co-expression correlations as low as ∼0.5 were identified in the “Overall” transcriptional landscape, but the coefficients of those confirmed across all 6 landscapes tended to be higher than this. This approach yielded a landscape with 3,506 nodes and 6,506 edges (Figure 3; and in more detail on the web site; http://www.livestockgenomics.csiro.au/courses/Hudson_muscle_transcription.html). Of the 6,506 edges, only 224 (i.e. 3.4% of the total) were negative (Table 1). This is consistent with [12] who computed a gene co-expression network across many human tissues and reported an over-representation of positive associations. The network and node file information for assembling the Always Correlated transcriptional landscape are in Tables S3 and S4 respectively.

**Table 1:** Network connectivity.

	Overall	Pied	Wagyu	PreNatal	PostNatal	Diets
**Overall**	22.92^1^	48.53%^2^	46.60%	27.08%	7.09%	3.49%
**Pied**	37.21%	16.55	39.99%	30.42%	7.27%	2.99%
**Wagyu**	37.95%	29.89%	18.74	27.98%	7.50%	2.88%
**PreNatal**	20.09%	22.92%	19.37%	13.20	4.20%	2.56%
**PostNatal**	1.78%	2.10%	1.99%	0.98%	5.87	3.06%
**Diets**	1.29%	1.14%	1.09%	0.90%	0.95%	3.03

1 Clustering coefficient (diagonal) for each network.

2 Percent overlap computed from the ratio of common links divided by the total number of unique links for positive (above diagonal) and negative (below diagonal) links across each pair-wise network comparison.

While a pair of genes in the Always Correlated landscape may be joined by a positive or negative edge, this does not imply that the significant correlation is the same sign in all six networks. While comparatively rare, there are several instances where the sign is positive in 5 of the 6 networks and negative in the other, and *vice versa* (yellow background in Table S3).

### Modules in the Always Correlated transcription landscape

The Always Correlated landscape comprised one large, cohesive network (2,620 nodes), two much smaller networks (65 and 18 nodes respectively) and a large number of very small networks containing 2 to 10 genes each. The biologically meaningful modules present in the main network could easily be discerned by eye once node colour was mapped to GO term, and were additionally verified by the *Cytoscape* plug-in MCODE (data not shown). Taking into account GO term and intra-connectivity, the major functional modules contain an enrichment of cell cycle, extracellular matrix, glycolysis/fast twitch muscle subunits, slow twitch muscle subunits, nuclear-encoded mitochondrial and mitochondrially-encoded mitochondrial genes. This enrichment was formalised statistically using the Cytoscape plugin, BinGO [30] after highlighting the key modules manually and selecting the “Biological Process” option for *Bos taurus*. The enrichments for the cell cycle, nuclear-encoded mitochondria, extracellular matrix and glycolysis modules were awarded the following P-values: 2.93 e^−13^, 1.10 e^−11^, 9.97 e^−6^ and 7.88 e^−15^ for “M-phase”, “oxidation reduction”, “collagen fibril organisation” and “cellular carbohydrate process”, respectively.

Furthermore, several mitochondrially-encoded mitochondrial genes (ND1, ND3, ND4, ND4L, ATP8) form a small cluster between the glycolysis and nuclear-encoded mitochondrial gene modules, indicating a spatially arranged ‘metabolic axis’ running coordinately through the main network. When including networks outside of the main networks, other functionally coherent modules included genes encoding neural, immune system, microvasculature, fat metabolism and ribosomal proteins. The ribosomal module comprised only 7 genes, much smaller than in previously published co-expression reports. This is because the editing procedure removed many genes encoding ribosomal proteins from the downstream analyses because of their low standard deviation. The small isolated network at the top left hand side of the overall landscape comprising 65 genes defied a simple annotation.

### Muscle contractile subunits in the Always Correlated transcriptional landscape

The positions of the bovine orthologues of the human and mouse fast, slow and embryonic muscle fibre type specific structural subunits were determined (Table 2). The majority (i.e. 7 of the 11 slow subunits, 7 of the 12 fast subunits and 2 of the 3 embryonic subunits) made it onto the network, and clustered in a manner consistent with their known biology. TPM2, not listed as a slow twitch fibre protein in [31], is located in the slow twitch module. MYH8, an embryonic myosin isoform, is located in the fast twitch module. However, it is negatively correlated with the other genes in the module, reflecting its' downregulation concurrent with the developmental upregulation of the fast twitch subunits. The genes encoding the fast twitch proteins lay in a module with a group of genes encoding proteins involved in glycolysis. In contrast, the slow twitch module did not contain any metabolic enzymes. In addition to the two large modules containing genes encoding muscle structural proteins, a number of smaller modules or clusters of genes also contain a subset of muscle structural proteins (Table 2). Interestingly, three small heat shock proteins, HPSPB3, 7 and 8, implicated in muscle function and myopathies [32] have expression patterns correlated with muscle structural proteins. HSPB1 is also in the Always Correlated landscape linked to HSPB8, to which it is also known to bind [32].

**Table 2:** Composition of modules containing muscle subunits in the Always Correlated network.

	Slow twitch fibres	Fast twitch fibres	Embryonic fibres	Other structural protein genes	Other muscle protein genes
Slow twitch module	MYL2, TNNT1, MYBPC1			TPM2, LDB3	MB, CA3, SH3BGR
Fast twitch module		MYH1, TNNT3, MYOM2, TPM1, ACTN3, MYBPC2, TMOD4	*MYH8*	NEB (tv), MYPN, SSPN	RYR1, ALDOA, ATP2A1, ENO3, CKM, PFKM, FBP2, PGAM2, DHRS7C, JPH2
In another module/cluster	[MYL6B, (HSPB3)] [TNNC1 (TRDN)] [TPM3 (HDAC3)]		[MYL4 (TMSB10, TMEM204, TUBB2B, *DLAT*)]	[MYBPH, MYBPHL[ [TTN (tv), NEB (tv)] [LMOD2, TTN (tv)] [SMPX (PDE4DIP)] [MYOT (GHITM)] [MYBPC3 (SFRS7)] [KBTBD5 (RPS6KA3, HSPB8)] [TNNT2 (TCF7L2, CTNNB1, NAV3, PSRC1, SH3PXD2A)] [TCAP, PDLIM3, (HSPB7)] [TRIM63, (SLC7A8)]	
Not in the Always Correlated network	MYH7, MYL3, MYOZ2, MYOM3, TNNI1, TMOD1	MYL1, MYH2, MYH4, TNNC2, MYOZ1, TNNI2, MYLPF	MYH3, MYL7	Many other genes	Many other genes

Fibre type assignments are from [31], except for TMOD1 and TMOD4 [64] and MYL6B [65].

*italics* negatively correlated with the majority of the members of the module.

tv – transcript variant.

[] module or cluster.

() non-structural protein.

### Hubs in the Always Correlated transcriptional landscape

Major hubs (i.e. the most highly connected nodes) in the network include two genes from the cell cycle module (DSE, DLGAP5) with 37 connections each. To formalise whether hub genes tended to belong to a particular gene ontology, we sorted the nodes by connectivity (in descending order) and the GO terms of those enriched at the top of the list was determined using the GOrilla tool [33]. The cell cycle was the top hit followed by glycolysis and cell adhesion. None of these fundamental cellular processes are specific to muscle tissue and not surprisingly the correlations transcend any muscle specific process. Conversely, muscle-specific genes were not enriched by this analysis. This information is displayed on the network using the more traditional connectivity criterion (Figure 3B).

Among the top 660 (10%) most connected genes in the Always correlated Landscape, there were 70 TF, implying an over-representation hypergeometric test p-value of 8.66E-6. This indicates that highly connected genes are more likely to be TF than would be expected by chance, at least in this PCIT-driven network. This phenomenon could be attributed to the partial correlation approach capturing causal connections [34]. However, given the canonical pro-myogenic TF are poorly connected or absent, we do not believe that connectivity in a co-expression network should be used as a simple proxy for regulatory importance.

### Effect of changing the correlation coefficient cut-offs

The coherency of the modules can be appreciated by increasing the correlation cutoff of the landscape construction. By focussing on only those connections with correlation coefficients greater than 0.99 one can construct a small landscape of 467 nodes and 644 edges (Figure 3C). This smaller landscape contains modules of cell cycle genes, glycolysis genes and genes encoding mitochondrial proteins, mirroring some of the main modules in the Always Correlated parent landscape and highlighting those modules built of only the most extreme correlation coefficients. The dynamic changing topology of the Always Correlated network can be visualised by changing the correlation cut-off incrementally. This is illustrated on Figure S1 which shows in 12 consecutive panels the topology of the network at the following thresholds: 0.75, 0.80, 0.85, 0.90, 0.91, 0.92, 0.93, 0.94, 0.95, 0.96, 0.97 and 0.98 respectively.

### Developmental expression profiles of the core modules

**Figure 4:**
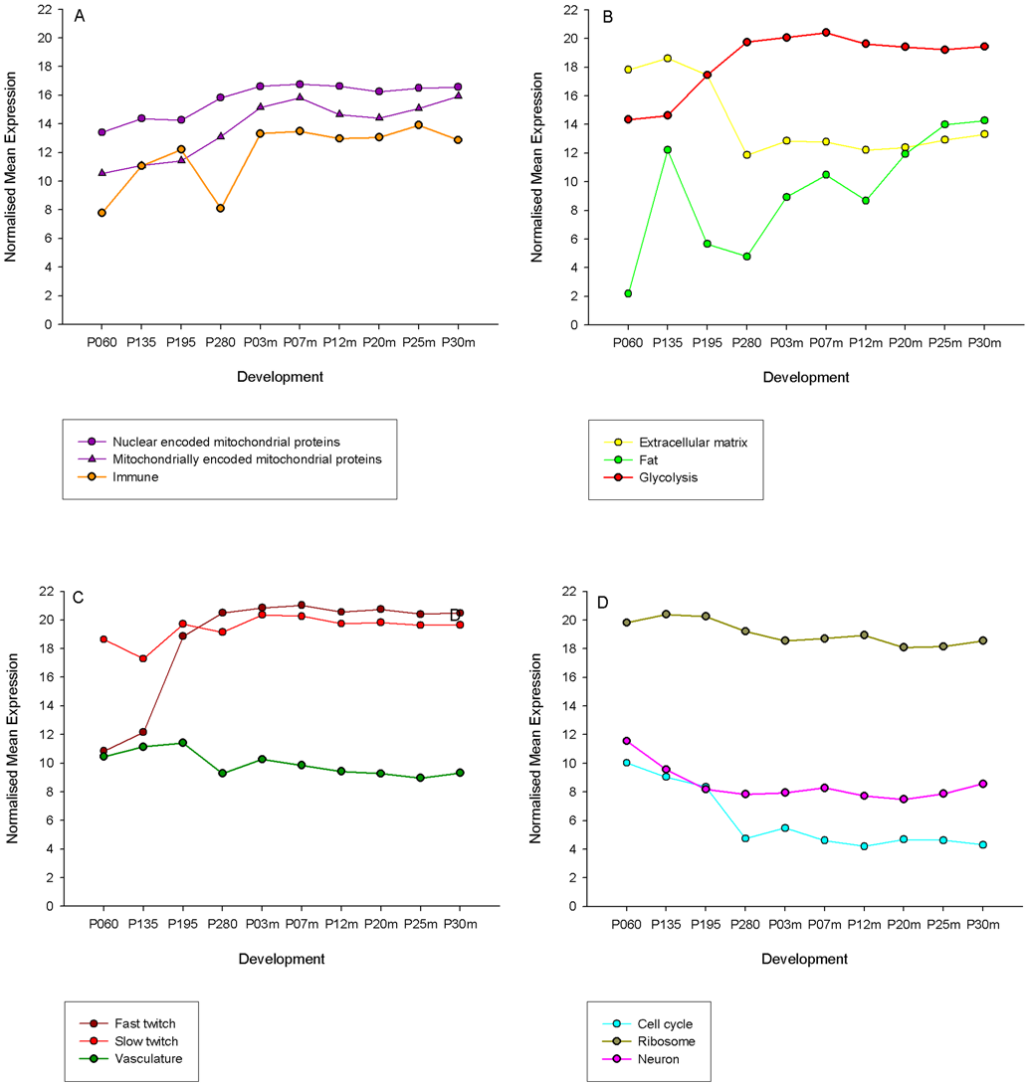
The expression profiles of mammalian muscle over development. Representatives from each of the main functional modules are shown: Immune, nuclear and mitochondrially-encoded mitochondrial genes (A); Extra-cellular matrix, fat and glycolysis gene transcription (B); Vasculature, fast and slow twitch muscle (C); and Cell cycle, ribosome and neuron gene transcription (D).

The consensus expression profiles of the core modules were plotted across the development time course for the Piedmontese×Hereford samples (Figure 4). Very similar profiles were observed for the same modules from Wagyu×Hereford animals (data not shown). The gene expression in the cell cycle and extracellular matrix modules are high in the prenatal samples and decline in the day 280 sample to lower post natal levels. In contrast, the nuclear and mitochondrially-encoded mitochondrial genes both show an increasing trend in expression level across development. Fat gene transcription is negligible at primary myogenesis but markedly induced by secondary myogenesis. Conversely, glycolysis gene expression ascends during pre-natal development and remains constitutively high in adult muscle. Fast twitch muscle transcription is much lower than slow twitch muscle transcription at primary myogenesis but by differentiation little difference in levels of gene expression between the fibre types are apparent. Other than birth which experiences a 3–4 fold down regulation, vasculature expression is moderate and fairly stable across all time points. The cell cycle gene expression crashes at birth coincident with the large-scale exiting of the cell cycle and transition to a post-mitotic state in mature muscle. As expected, ribosomal gene expression is high and stable across all time points.

### Transcriptional Regulators in the Always Correlated transcriptional landscape

**Figure 5:**
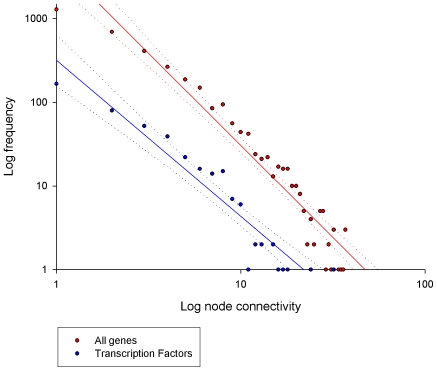
The connectivity of all genes in the Always Correlated transcriptional landscape versus the transcriptional regulators.

As the network is scale-free (indicative of being non-random) the vast majority of nodes have very few connections. This observation applied to both TF (of which there were 430, of the 1,017 in the list, included in the networks) as well as all genes (n = 3,506) (Figure 5). In addition to the TFs, 23 chromatin remodelers and 405 signalling molecules were also include in the networks (Table S1). In some instances, the regulators of a given module will make it into the module itself based on the PCIT networking strategy. For example, ESRRA – a recently discovered regulator of mitochondrial biogenesis [35] - is a member of the mitochondrial co-expression module. Equally, SOX17 – a known major regulator of vasculogenesis [36] – is a member of the microvasculature module. The transcriptional regulators present in each of the major modules are documented in Table 3. This shows that the PCIT-driven co-expression method can, in many circumstances, cluster regulators with their targets in an unsupervised fashion, and underscores the ability of weighted networks to identify causal relationships.

**Table 3:** The transcriptional regulators correlated with the functional modules.

Module gene ontology	Transcription Factors common to both approaches	Transcriptional Regulators present in the Always connected landscape modules and not in Top 10^1^	Top 10 correlated Transcription Factors in the Overall landscape using the Module-to-Regulator analysis^2^
Cell cycle	FOXM1 [66], E2F8 [67], E2F1 [68], E2F2 [69], HELLS [70]	No TF/NDC80 [71], TOP2A [72]/no chromatin	E2F1, CBX2, TCF19, MYBL2, FOXM1, CDCA4 [73], HELLS, E2F2, E2F8, TIAM1 [74]
Glycolysis/Fast Twitch	none	EP400, ZBTB7A, MAFB, CBX8, SIX1 [51], TBX15, YBX1, ELF3/SHC1 [75], OR5A1, LGR4, PDE4C, ASB12, PTPLA, GPR98, GPR83/SETMAR	BGLAP, TBX15, PAX2, HLF [54], TADA2L, NPAS3, ARNT2, CHD3, GTF2IRD1 [58], ZNF521
Mitochondria (nuclear-encoded)	none	ESRRA [35], MAX, HIF1AN, SMARCB1, RNF14, SMARCA4, MBD1, TAF6, ZNF583, MYF6, ZNF618, EBF2/BSG, HOMER2, C5ORF13, WSB1, STYXL1, GRM2/no chromatin	CEBPB [76], PIR (co-factor of NFI, see [77]), CDCA7, NR4A1, ZNF358, ZBTB7B, TFB2M [37], CBX2, KLF9, AFF1
Mitochondria (mito-encoded)	none	EBF3, PAWR/EFNA2, SMURF1, ADRBK2/no chromatin	LBX1, ZNF358, ATF4, THRB, NFIX, BHLHB3, BGLAP, CREB3L4, GPS2, ZBTB7B
Extracellular matrix	PHF19	PCBD1, PBX3/CARHSP1 DCLK1, ANGPTL1 [78], ELMO1, GEM, OPHN1, CNIH, S100A10, VAV3, LTBP4, ANK2, IQGAP2, SPARC [79], IGFBP6 [80], DDR2, GPR124, TRAF3/no chromatin	TIAM1 [81], PHF19, SOX12, CBX2, CDCA7, SREBF2, MYEF2, E2F2, TCEAL8, CDCA4
Immune system	IRF1 [57]	none	IRF1, RNF14, TEAD1, LRRFIP1, EPAS1, NR1D2, RORC, PCGF5, PHF12, HOXD8
Microvasculature	SOX17 [36], SOX18 [36], TAL1	none	SOX17, HES2, FOXF1, TAL1, SOX18, LHX6, TCF7, LMO2, NRIP2, ZHX1
Ribosomal proteins	none	none	SCMH1, CITED1, RBM4, ILF2, PRDM16, PRMT1, DPF3, NCOA5, CDCA7L, TRIM28
Fat	none	none	TAF6L, DRAP1, ZNF219, ZNF496, CITED1, PIAS1 [55], [56], HIF1AN, PAX1, RLF, MTERFD3
Neural	TLX3 [38]	ZNF621/ACCN2, AKAP7/no chromatin	TLX3, IRX6 [82], LHX1 [83], HOXD10 [84], DLX5 [85], HES5 [86], BCL11A [87], MEIS2 [88], HOXB3 [89], SSBP3 [90]

1 Order = TFs followed by Signalling molecules then Chromatin remodellers with “/” separating the 3 groups.

2 Order = descending strength of absolute average correlation coefficient. References providing experimental evidence supporting our computational output are provided.

However, while regulators in general perform similarly to all the genes in the landscape in terms of their connectivity distribution (Figure 5), we noticed that many of the most fundamental transcriptional regulators, including the canonical pro-myogenic TF themselves, are often poorly connected, or indeed completely absent from the network. This observation impacts on our ability to correctly reverse-engineer transcriptional regulation using basic co-expression approaches. For example, MEF2C and MYF5 are totally unconnected and fail to make it onto the landscape, while MEF2A, MEF2B, MYF6, MYOD1 and MYOG are connected to one gene only, namely KPNA3, GSTK1, FHOD1, MYOG and MYOD1, respectively. ANKRD1, ANKRD2 and CSRP3, muscle-specific transcriptional modifiers, also did not make it into the Always Correlated landscape.

### Module-to-regulator analysis

In an attempt to identify more of the major regulators of core biological processes purely with co-expression analyses we took advantage of the computed topology of the Always Correlated landscape to amplify the signal to noise for a subsequent downstream analysis. We computed the average absolute co-expression of *bona fide* TFs (from the conservative TF list) to those genes present in the functionally coherent modules identified in the parent network (refer to Table S2 for input target gene lists i.e. the genes present in a given module). The computation of the absolute values, versus the ‘signed’ values, is clearly an important modification when modules are connected by mixed signs. However, in reality the vast majority of co-expression network connections are positive. To compute the module-to-regulator correlations we used the values obtained from the ‘Overall’ network. A number of known regulators were identified by the Module-to-Regulator analysis which were absent from the PCIT-driven network approach (refer to Table S5 for the full output). TF with a large number of connections to a module (i.e. hubs) are also more likely to be awarded a high ranking (specific to that module) by the module-to-regulator analysis.

While we assessed the absolute, average correlation of all genes on the array, the *bona fide* TF were coded numerically so that their output could be specifically identified. Our discussion centres on the TF output specifically. An example of the extra TF information provided by the Module-to-Regulator analysis is TFB2M, a fundamental regulator of mitochondrial transcription [37] which has very high average absolute correlations (0.92 and 0.89) to genes in both the nuclear-encoded and mitochondrially-encoded mitochondrial modules, but does not make it into either module by PCIT because no single connection is deemed significant enough.

**Figure 6:**
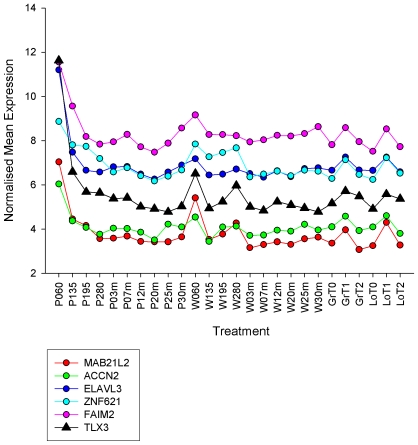
The expression profiles of the neuron module genes across the Overall landscape (i.e. the 10 Piedmontese and 10 Wagyu development time points, plus the starvation-realimentation experiment). The expression profile of the neurogenesis TF TLX3 is also shown, which did not make the module by PCIT but was ranked top by the downstream (nerve) module-to-regulator analysis.

Table 3 provides a synopsis of the main results for each module, comparing the two methods. For the purposes of illustration, the co-expression profiles of the genes present in the neuron module, plus that of TLX3 [38] (which was not a member of the PCIT-driven module, but was identified by the Module-to-Regulator analysis) are shown in Figure 6 using the 26 treatments of the Overall landscape.

## Discussion

In this paper we present by far the most comprehensive mammalian skeletal muscle co-expression network to date. It is based on the muscle expression profiles of 6,603 variably expressed bovine genes, assembled using a data-driven information theoretic based algorithm called PCIT [24]. The exceptional utility of information theoretic approaches in reverse-engineering networks has recently been formalised in a competitive arena. In the DREAM2 genome scale network challenge the various competing algorithms were presented with a compendium of 500 normalised *E. coli* microarrays [39]. The winner was the CLR algorithm of [40] which, in an analogous fashion to PCIT, “goes beyond the pairwise mutual information to include the state of a third gene…” [39]. The combination of the Module-to-Regulator analysis with PCIT correctly inferred a number of components of the regulation of biological processes that are conserved across the experimental perturbations. The success of the module-to-regulator analysis suggests that our ability to compute transcriptional regulation was augmented by using the topology of the co-expression modules. This input data enriches for the conserved connections and generates a less noisy set of target genes for subsequent downstream analyses.

**Figure 7:**
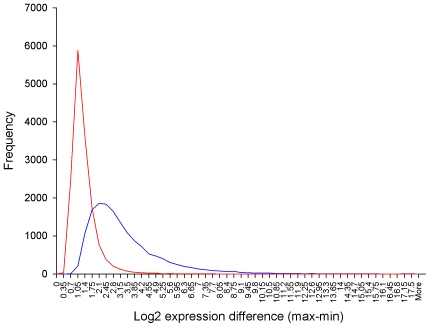
Range in expression level of genes versus frequency. Distribution of genes in postnatal Piedmontese and Wagyu samples in red and in all Piedmontese and Wagyu samples in blue. Including pre-natal as well as post-natal muscle stages increases the exploration of parametric expression space. An increase in the frequency of genes experiencing moderate-high changes in expression level reduces the formation of spurious edges in the computed co-expression networks.

Our experimental design leads to a highly robust co-expression transcriptional landscape. This is because the biological perturbations are so dramatic they force more genes to aggressively span a high proportion of the parametric space for the expression signals (Figure 7). While experimental perturbations are not a necessary condition for inferring co-expression networks [41] , they are considered useful [42] and will influence the topology of the landscape. In this respect, the foetal developmental perturbations are particularly discriminatory because pre-natal bovine muscle undergoes proliferation followed by differentiation, through two (or three) major waves of myogenesis. The changing cellular composition of the tissue helps remove spurious correlations and ensures only the most fundamental relationships remain. Since some modules are more robust than others in terms of their average correlation coefficients (Figure 3C) the impact of a given set of experimental perturbations may not be even across the landscape.

As a whole, the co-expression landscape is highly modular (Figure 3A) and scale-free (Figure 5) [11]. These observations are consistent with real biological regulatory networks. However, unlike real regulatory networks which tend to be disassortative, i.e. the hubs are not linked [11], we find that the muscle co-expression networks are more assortative (e.g. there are a number of inter-connected hubs). The highly assortative nature of the Always Correlated landscape is clearly evident when node size is mapped to connectivity instead of transcript abundance (Figure 3B). From this perspective, the large nodes are clustered together, particularly in the cell cycle, extracellular matrix and glycolysis modules. This assortativeness reduces the extent to which the co-expression landscape can be considered an “ultra-small world” since most nodes are not near a hub.

On the other hand, mapping node size to transcript abundance (Figure 3A) is appealing because it emphasises those proteins that make major structural contributions to the tissue. From this visualisation perspective, it is clear that the major components of skeletal muscle, irrespective of each treatment, are the various muscle contractile subunits, the mitochondria, and the extracellular matrix. Because so many of the biopsies were derived from adult muscle which is post-mitotic, the cell cycle module correctly appears small and insignificant (despite its exceptional cohesiveness). The overall modular resolution we observe in our bovine muscle network strongly resembles the co-expression output of [43], a similar analysis based on a comprehensive set of 24 healthy human tissues. The major modules discovered by [43] were nuclear-driven metabolism, ribosomal proteins, mitochondrial metabolism, immune response, metal ion homeostasis, extracellular matrix and cytoskeleton.

The modules identified by the analysis are important because they reflect fundamental structural and functional components of skeletal muscle biology. For example, the nuclear- and mitochondrially-encoded mitochondrial genes determine the physiology of the mitochondria, the sub-cellular organelle where bioenergetic conversion takes place. Any capacity to infer the transcriptional regulation of this module alone has downstream implications for changes in mitochondrial performance. In turn, this information may help elucidate the role of mitochondrial physiology in mammalian evolution [44], thermoregulation [45], cell and organismal senescence [46], athletic performance [47] and disease states such as the metabolic syndrome [48].

Skeletal muscle would appear *prima facie* to be an amenable tissue for co-expression analysis as its anatomy is strongly hierarchically organised from z-disc to sarcomere to myofibril to muscle fibre to whole muscle [49]. This organisation is partially reflected in the strong co-expression of a sub-set of the component molecules in the Always Correlated landscape. For example, we observe a highly connected module comprising a range of fast muscle structural subunits (MYH1, TNNT3, MYOM2, MYBPC2, TMOD4, ACTN3 and TPM1), along with the glycolytic enzymatic machinery. In accordance with [17] the slow twitch modules tend to be somewhat less coherent although MYL2, TNNT1, MYBPC1, TPM2 and MB were clustered. The absence of some of the expected fast and slow subunits, and the scattered distribution of many of the other genes encoding muscle structural proteins suggests that even within a single muscle there is a less discrete, and more continuous, range of muscle fibre compositions at the anatomical level. This continuum presumably satisfies various developmental, evolutionary and environmental circumstances. A more detailed analysis of the combination of treatment transcriptional landscapes with the output modules is required to tease out the subtleties regarding the relationships between the muscle structural proteins.

We used the co-expression modules to further examine key expression profiles across bovine muscle development to form what may be called ‘expression motifs’ for the most cohesive well-annotated modules (Figure 4). This approach is somewhat equivalent in concept to the module eigen-genes of [50] in the sense that we are highlighting representative traces that capture the main ‘transcriptional behaviours’ across mammalian skeletal muscle development. Of these core motifs, the most variable across the 10 developmental time points are the 1) cell cycle and extracellular matrix (which are both high pre-natally and crash at birth), fast twitch subunits which are low prenatally but rise markedly at birth, and 2) fat, which rises markedly coincident with secondary myogenesis before dropping again, and then rising first at 7 months postnatal (coinciding with the appearance of visible intramuscular fat) followed by a further rise at 25 months. The cell cycle pattern reflects the active cell proliferation that occurs pre-natally in mammals when muscle fibre number is determined, and the crash coincides with a coordinated large-scale exiting of the cell cycle prior to birth; adult skeletal muscle being post-mitotic in mammals, apart from a small population of satellite cells.

### Transcriptional regulators

In some cases the master regulators of a given module were components of the relevant module, based purely on the PCIT driven network analysis. Examples include, ESRRA [35] in the mitochondrial module, SOX17 [36] in the microvasculature module and SIX1 in the glycolysis/fast twitch module. Forced expression of SIX1 and EYA1 in slow twitch mouse *soleus* muscle has been shown to induce a fibre transition characterised by the replacement of myosin heavy chain I and IIA isoforms, the activation of fast twitch fibre-specific genes and a switch towards glycolytic metabolism, providing clear experimental support for our basic network output [51]. In the study of [5] EYA1 was identified as one of the key next level TF in the regulatory cascade initiated by MYOD1. In our study EYA1 was not identified by any of the analyses. However, EYA1 interacts with and modifies the activity of SIX1, which was identified in our analyses. Consistent with the model of [5], four of the six genes that they identified as regulated by EYA1/SIX1 were members of our glycolysis/fast twitch module (the other two did not make it into the Always correlated network) to which SIX1 was linked. Interestingly, EYA1 did not make it into the Always Correlated landscape and unlike SIX1, which has approximately four fold increased expression during prenatal development and little change in post natal development, EYA1 expression decreases approximately four fold during prenatal development, increasing post-natally to approximately the same level as day 60 prenatal samples.

Our new downstream analysis computes the absolute average correlation of expression of TF across a set of genes, which themselves were identified by inclusion in a co-expression module of interest. This approach is highly analogous to [52] who generate bootstrap replicates of a module and locate the TF that correlates the strongest across the replicates. Our version of this approach performs well, presumably because it amplifies the signal to noise ratio (as it is not reliant on a significant connection to any given gene). The new output can then be ranked according to correlation coefficient, and we find the that list of best candidates (i.e. average correlation coefficients close to unity) is enriched for known TF of many of those processes (Table 3).

Using this data-driven approach, in conjunction with the conventional PCIT-driven co-expression output documented above, we correctly inferred known regulators cell cycle activity (e.g. E2F1 [53]), glycolysis (e.g. HLF [54]), mitochondrial transcription/biogenesis (e.g. TFB2M [37]), adipogenesis (e.g. PIAS1, circumstantial evidence only [55], [56]), neuronal development (e.g. TLX3 [38]), immune function (e.g. IRF1 [57]) and vasculogenesis (e.g. SOX17 [36]), all within a skeletal muscle context. We also discover that GTF2IRD1 ranks highly against the fast twitch muscle module. Like SIX1, GTF2IRD1 has been shown to culminate in a complete loss of slow twitch fibres and concomitant replacement with fast type IIA fibres under transgenic conditions [58].

### The promise and limitations of co-expression networks

The approaches documented above generated a host of new candidate regulators for these biological processes, several of which have unknown functions and represent excellent candidates for future wet-lab validation. It appears that, depending on circumstances, both PCIT and the Module-to-Regulator analyses perform well in reverse-engineering known regulatory biology. The most robust predictions are presumably for those regulators common to both analyses (Table 3 column 1). A challenging overall outcome was that in the specific cases of the well-known muscle fibre type composition regulators PPARGC1A [59], PPARD [60], MSTN [61] and AKT1 [62] neither PCIT nor the Module-to-Regulator analysis performed convincingly in aligning them with the anticipated muscle module (refer to Table S5 for full regulator-to-module output). Similarly, the canonical pro-myogenic muscle gene TFs (MRFs), such as MYOG, MYOD1, MEF2C, MYF5 and MYF6 were not identified in either of the analyses as correlated with the expression of genes encoding muscle structural proteins. MYOD1 regulates MYOG expression and based on ChIP-on-chip studies [5], [6] these TFs are predicted to regulate many genes in common. In our analysis, they form a separate cluster of just the two transcription factors. The genes to which MYOD1 and/or MYOG bind in ChIP-on-chip experiments have a very wide range of functions, which may contribute to the lack of high correlation with the functional modules observed in the data. It has been reported that TFs are the largest cluster of MRF targets, implying that there may be an extensive regulatory cascade from the MRFs to the genes encoding muscle proteins [5]. Arguably, the longer and more complex the regulatory cascade between TF and target gene, the less likely they will be highly correlated to each other.

In part the relative lack of muscle structural gene modules will also have contributed to the lack of association between the MRFs and the genes encoding the muscle structural proteins. However, further to this TF do present special challenges to co-expression analyses: 1) TF tend to be expressed at basal levels close to the sensitivity of high-throughput technologies [34] whereas their targets are often abundant and variably expressed 2) TF often control their targets combinatorially and so their own co-expression relationship is complicated by the performance of their regulatory partner, 3) TF activity is frequently independent of its own expression level. Thus, even in the absence of a change in expression level, a TF can be strongly activated by ligand or co-factor binding, phosphorylation, translocation to the nucleus, and the formation of transcriptionally open euchromatin and 4) TF can have different functions at different stages of development. Together, these mechanisms tend to break expression-based correlations between TF and their targets.

In light of these complications, a powerful conclusion can be drawn about regulators who successfully make it into the expected module (for example ESRRA in the mitochondrial module and SOX17/18 in the vasculature module). The implication is that - at least across the treatment contrasts used in the computation of the network - they experience no change in regulatory partner, no change in ligand binding, no change in phosphorylation state, no reversals in behaviour relative to developmental stage and no change in cellular localisation that significantly affects their activity. Put another way, all of their change in regulatory activity may result from simple changes in their own expression level. Such regulators readily lend themselves to basic co-expression analyses; they are the ‘low-hanging fruit.’ But what of those regulators who are activated in more complicated and subtle ways - are there methods for identifying them through gene expression data alone?

In our data there is a notable case study. Myostatin, a negative transcriptional regulator of muscle development is absent from the Always Correlated landscape and poorly correlated to the fast (0.33) and slow (0.26) twitch modules. Myostatin harbours a missense SNP in the Piedmontese animals [63] but not the other breeds we sampled. Despite no change in transcript level, the myostatin SNP leads to the translation of a dysfunctional protein that is less able to put the brakes on Piedmontese fast twitch muscle development [10] which increases their muscularity relative to the other breeds. How do we reconcile the poor correlation of myostatin to the fast twitch module on the one hand, with its accepted biological role in repressing fast twitch muscle development on the other?

In our opinion the deconvolution of molecules like Piedmontese myostatin, whose change in regulatory behaviour is manifest almost exclusively post-transcriptionally, requires more sophisticated methods than simply identifying high co-expression coefficients to functional modules. One such method is the calculation of Regulatory Impact Factors computed across the appropriate experimental contrast - in this case the myostatin mutant breed versus a wild-type breed [10].

These various regulatory complications thus highlight an interesting asymmetry in the interpretation of the analysis. While one can be reasonably confident that a regulator present in a co-expression module plays a role in that module, one cannot safely make the converse conclusion. In other words, it is not permissible to conclude that a regulator absent from a module does not contribute to its regulation.

As Aaron Levenstein might well have said, “what co-expression networks reveal is suggestive, but what they conceal is vital.”

## Supporting Information

Figure S1 The changing topology of the Always Correlated landscape as the correlation cut-off is made increasingly stringent. The ‘metabolic axis’ is clearly preserved in most of the networks, despite other large-scale shifts in orientation and topology. The same main modules are present in all but the most stringent of the networks.
(0.59 MB TIF)

Table S1 The list of transcriptional regulators used to map the network based on bona fide DNA binding TF, plus text searching GO terms for “chromatin,” and “signal.”
(0.04 MB XLS)

Table S2 The genes present in each Always Correlated module.
(0.03 MB XLS)

Table S3 The Cytoscape Always Correlated landscape file.
(1.31 MB XLS)

Table S4 The Cytoscape Always Correlated Node file.
(0.56 MB XLS)

Table S5 The module-to-regulator analysis i.e. the absolute, average correlation of all genes on the array to each Always Correlated module. The six digits after each gene name correspond to confirmation (1) or rejection (0) of whether the gene is a TF, post-translational modifier, kinase, secreted, methylated and alternatively spliced. The TF output forms the basis for the majority of our discussion. The other annotations are provided as a systems resource for researchers who may have specific interests in those properties.
(9.40 MB XLS)
