# Supplementary figures and images for: Inferring the Transcriptional Landscape of Bovine Skeletal Muscle by Integrating Co-Expression Networks
Source: PLoS One. 2009 Oct 1;4(10):e7249. doi: 10.1371/journal.pone.0007249 (PMC2749936; doi:10.1371/journal.pone.0007249)

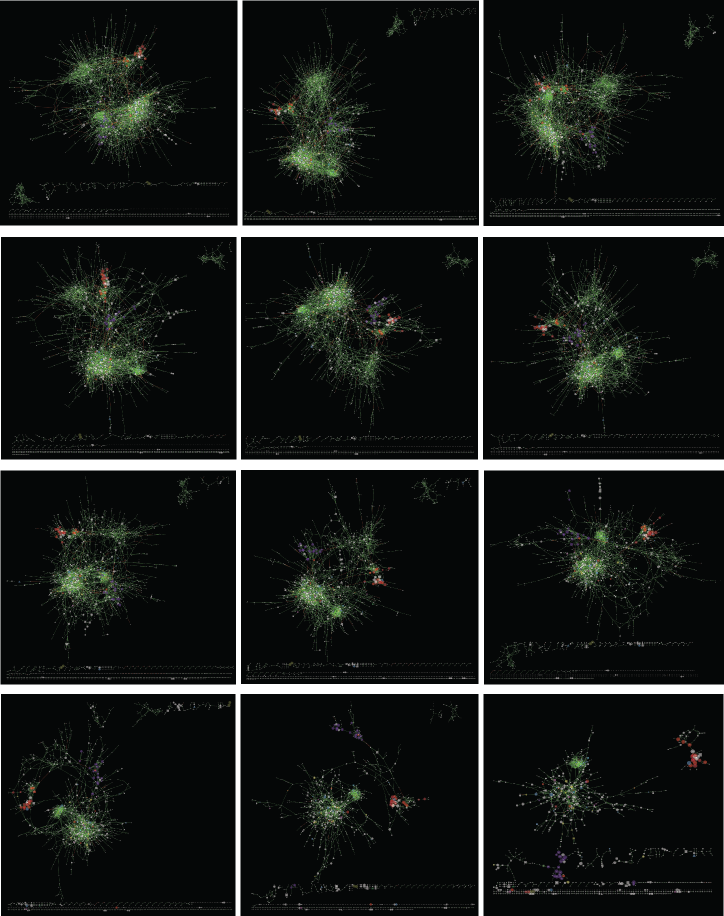

Supplement: Figure S1 — The changing topology of the Always Correlated landscape as the correlation cut-off is made increasingly stringent. The ‘metabolic axis’ is clearly preserved in most of the networks, despite other large-scale shifts in orientation and topology. The same main modules are present in all but the most stringent of the networks. (0.59 MB TIF) [file pone.0007249.s001.tif]
